# Effects of Multiple Judo Bouts in the Bilateral Strength Deficit on the Countermovement Jump Metrics

**DOI:** 10.5114/jhk/202147

**Published:** 2025-09-23

**Authors:** Rafael Lima Kons, Jonathan Ache-Dias, Filipe Estácio Costa, Juliano Dal Pupo, Daniele Detanico

**Affiliations:** 1 Department of Physical Education, Faculty of Education, Federal University of Bahia, Bahia, Brazil.; 2 Biomechanics Laboratory, Center of Sports, Federal University of Santa Catarina, Santa Catarina, Brazil.; 3 Research Group on Technology, Sport and Rehabilitation, Catarinense Federal Institute, Araquari, Santa Catarina, Brazil.

**Keywords:** muscle imbalance, combat sports, vertical jump, vertical impulse, martial arts

## Abstract

In official judo competitions, athletes often participate in multiple bouts with short recovery intervals, leading to progressive fatigue that impacts neuromuscular performance. This study examined the acute effects of four simulated judo bouts on bilateral strength deficit (BLD) using metrics from countermovement jumps (CMJs). Thirteen male national-level judo athletes (21.4 ± 2.9 years) participated in the study. Athletes performed both unilateral and bilateral CMJs in randomized order: first at baseline before the initial match, and then after each of the four subsequent matches. The CMJ metrics evaluated included peak and mean power, jump height, peak force, peak velocity, and vertical net impulse. The study used the typical error (TE) and the intraclass correlation coefficient (ICC) to assess relative and absolute reliability. To compare CMJ metrics across the matches, the Friedman test with Conover’s post hoc analysis was conducted, with statistical significance set at p < 0.05. The main findings showed excellent relative reliability, with an ICC greater than 0.85 for all variables in both limbs. Significant effects of the judo matches were observed only in the CMJ impulse (which decreased after the third match) and in BLD of impulse, which increased after the third match (p < 0.05). No statistically significant changes were found in other CMJ metrics. These results suggest that while successive judo bouts induced significant fatigue and force deficits, detectable through changes in impulse, other CMJ metrics remained unaffected. This may indicate that the capacity to generate force quickly in the lower limbs is primarily impacted by successive judo bouts.

## Introduction

In official judo tournaments, athletes typically participate in several bouts, usually ranging from four to five, each lasting four minutes with the possibility of an unlimited extension (e.g., golden score). These bouts are separated by intervals of 10 to 15 min (Detanico et al., 2015; Franchini et al., 2003). During each bout, judo athletes engage in high-intensity tasks, with vigorous efforts lasting, on average, 20–30 s, interspersed with 7–10-s pauses (Franchini et al., 2013). As a result, the accumulation of fatigue throughout real competition is inevitable (Detanico et al., 2015). Muscle fatigue generally leads to altered muscle function (Galán-Rioja et al., 2024; Lyu et al., 2025), which can negatively impact physical performance metrics. A recent systematic review highlighted that performance in both the lower and upper extremities was impaired by repeated bouts in combat sports, as evidenced by declines in handgrip strength and vertical jump performance (Kons et al., 2020). Specifically, in the lower limbs, a 3.6% decrease in jump height was observed after the second bout, followed by a 3.2% decline after the third bout in a simulated tournament (Detanico et al., 2015), indicating that athletes experienced progressive fatigue during the combats. This issue is particularly relevant for judo athletes (Kons et al., 2020), especially when considering strategies to mitigate fatigue and preserve neuromuscular performance throughout competition.

The critical role of the lower extremities in judo tasks is well-established (Franchini et al., 2011), evidenced, for example, by the correlation between performance in jump height and judo-specific actions (e.g., attacks and throws in specific tests) (Kons et al., 2018; Detanico et al., 2012), as well as overall combat effectiveness in official competitions (Kons et al., 2017). The buildup of fatigue due to repeated high-intensity efforts has been identified as a crucial aspect contributing to muscle imbalance, particularly the bilateral deficit (BLD) of strength (Kons et al., 2023), as found in judo-specific tasks (Turnes et al., 2022; Kons et al., 2021). BLD suggests that the force generated during a maximal bilateral contraction is less than the combined force produced by each limb when measured individually (Bishop et al., 2021; Bobbert et al., 2006; Botton et al., 2013; Bracic et al., 2010). According to Železnik et al. (2022), in ballistic contractions, such as jump assessments, BLD may indicate the difference between the height of a two-leg jump and the combined heights of one-leg jumps. It can also be derived from other jump metrics, including power, force or impulse. Although BLD can be described as a multifactorial phenomenon (i.e., physiological and neurophysiological factors), the prevailing explanation is interhemispheric inhibition, which refers to the suppression of activity in one cerebral hemisphere by the other (Anders et al., 2021; Škarabot et al., 2016; Železnik et al., 2022).

Previously, Kons et al. (2021) observed a significant increase in BLD obtained by the handgrip strength test after the second simulated judo combat, showing that the accumulation of fatigue affected the magnitude of BLD. Another study found that the magnitude of BLD obtained by the countermovement jump (CMJ) was negatively associated with judo-specific actions (e.g., performance in the Special Judo Fitness Test) (Kons et al., 2022). In this context, exploring BLD through CMJ metrics during a fatigue situation could offer valuable insights for judo coaches and sports scientists to make informed decisions regarding each athlete's conditioning status. The CMJ test is considered useful for assessing muscle fatigue, as it reflects the stretch-shortening capability of the lower limbs and causes minimal additional fatigue (Halson, 2014).

Considering this context, investigating muscle fatigue in judo is crucial due to the unique demands placed on athletes during official competitions, where they compete in multiple high-intensity bouts with limited recovery time (Kons et al., 2018). While prior research has documented performance declines in combat sports due to fatigue (Barbas et al., 2011; Bonitch-Gongora et al., 2012; Detanico et al., 2015; Kons et al., 2020; Kraemer et al., 2001), there is a notable gap in studies specifically examining the behavior of BLD during consecutive judo bouts. This study aimed to fill that gap by employing CMJ metrics, effectively assessing muscle function while minimizing additional fatigue. Therefore, the current study intended to analyze the effects of four successive judo bouts on BLD of the CMJ. We hypothesized that BLD would be present and progressively increase over the judo bouts.

## Methods

### Experimental Design

This research utilized an experimental design to examine the immediate effects of simulated judo bouts (a total of four) on BLD levels, assessed through the CMJ test. The simulation involved four judo bouts, each lasting four minutes, mirroring actual combat time, with 15-min passive rest periods in between. Athletes conducted both unilateral and bilateral CMJs in a randomized sequence, with assessments taking place at baseline before the first bout and immediately following each bout, also in randomized order. Furthermore, all participants took part in a familiarization session one week prior to the trials.

### Participants

Thirteen male judo athletes participated in the current study. Participants were 21.3 years old (± 3.0) and had an average of 16.7 years (± 3.6) of judo practice, with a range from 10 (minimum) to 20 years (maximum). All athletes competed at the national level. Their mean body mass was 70.4 kg (± 19.4), body height was 175.0 cm (± 5.2), and the body fat percentage was 12.6% (± 5.5). The sample size was calculated a priori through G*Power software (version 3.1.9.2., Universität Kiel, Germany) for ANOVA repeated measures, within-factors (effect size = 0.25, *p* = 0.05, β = 0.80, number of groups = 1, number of measurements = 1, correlation among repeated measures = 0.7, non-sphericity correction = 1). Based on these variables, a minimum of 12 participants per group was required. All athletes participated in national tournaments and trained regularly—both technically and physically—three to four times a week, holding either brown or black belts. The inclusion criteria were as follows: all participants had to demonstrate a consistent training regimen that included physical, technical, and tactical components, and train four to five times a week for the past five years. They also needed to be in a competitive phase, avoiding any periods of rapid weight loss. Participants were required to be over 18 years of age and provide written informed consent after receiving a detailed verbal explanation of the study’s purpose, methods, and potential risks and benefits. They were also asked to confirm that they had no musculoskeletal disorders or injuries in the year leading up to the evaluations and no physical restrictions that could affect maximal performance. Furthermore, athletes needed to abstain from alcohol and medication for at least 48 h before and during the evaluations, and adhere to their usual diet. Before the assessments, all participants were briefed on the procedures and signed an informed consent form. The study was approved by the Institutional Review Board of the Federal University of Bahia, Bahia, Brazil (protocol code: 5.714.111; approval date: 20 October 2022) and conducted in accordance with the Declaration of Helsinki.

### Judo Bout Simulations

Prior to the simulated bouts, athletes participated in a 15-min warm-up, which involved dynamic stretching for both upper and lower limbs and practicing judo-specific throws, such as o-soto-gari and seoi-nage (judo leg and arm-specific techniques, respectively). Following the warm-up, they competed in four (4-min) judo bouts (simulated), each followed by a 15-min rest period. Participants were required to complete all bouts (a total of four). If an ippon (highest point) was scored, the match would restart to ensure consistency in duration for all competitors. Opponents were paired based on a maximum 10% difference in body mass. This protocol effectively replicated the temporal structure of a real judo combat, as supported by relevant literature (Kons et al., 2018, 2020). The time between the end of each match and the following assessments ranged from six to eight minutes, allowing for seven to nine minutes of passive rest within the entire 15-min interval. This timing was established based on a pilot study and previous research (Kons et al., 2021), ensuring sufficient time for athletes to move to the local laboratory from the dojo, where the bouts were held. During the assessments, the temperature in both the laboratory and dojo (judo training place) was kept constant at 24°C.

### Countermovement Jump Assessment

Before testing, athletes engaged in a warm-up that included two sets of ten hops on the ground, followed by three to five submaximal countermovement jumps. They also performed four single-leg countermovement jumps for each limb (designated as dominant and non-dominant by athletes), along with the same protocols for bilateral assessments. After a three-minute rest period, participants were assessed in the countermovement jump (CMJ) in randomized order. The average of the three jumps was calculated to determine each variable.

Athletes were instructed to position themselves in the center of a piezoelectric force plate (Model 9290AD, Quattro Jump, Winterthur, Switzerland) with their arms akimbo to minimize arm involvement. To start the test, they lifted one leg off the ground, performed a countermovement, and then executed a rapid extension of the lower limb joints to jump as high as possible. Participants were encouraged to reach their maximum height using their preferred squat depth. They practiced maintaining consistent jump technique and knee flexion across all attempts. Each athlete completed three maximal unilateral and bilateral CMJs at baseline and immediately after each match. Based on power spectral analysis, the ground reaction force (GRF) data were analyzed using a fourth-order low-pass Butterworth filter at 20 Hz. From the force-time curve, the following variables were derived: 1) jump height (calculated through double integration of the GRF); 2) power output [peak and mean] (determined by multiplying the GRF by the velocity during the concentric phase of the jump); 3) peak force (representing the maximum value reached during the concentric phase); 4) peak velocity (the highest velocity recorded just before takeoff); and 5) net impulse (calculated by subtracting the vertical impulse due to gravitational acceleration) (Kirby et al., 2011). The trapezoidal rule was applied to approximate the integral of the force-time curve over the interval starting one second before the movement (ensuring zero velocity) and extending to the moment of the take-off.

### Bilateral Deficit of Strength

BLD was calculated as the ratio between bilateral and unilateral countermovement jump (CMJ) metrics. This calculation utilized the right/left unilateral and bilateral values, as illustrated in equation 1:


$$BLD\left(\%\right)=\left[100*\left(\frac{Bilateral}{left\;unilateral+right\;unilateral}\right)\right]-100$$

Bilateral facilitation was identified when BLD was significantly above 0, whereas bilateral deficit was noted when BLD was significantly below 0. Bilateral deficit or bilateral facilitation was defined as a meaningful difference between the performance in bilateral tasks and the combined results of the unilateral tasks (Howard and Enoka, 1991).

### Statistical Analysis

The indicators of absolute and relative reliability were assessed using the typical error (TE) and the intraclass correlation coefficient (ICC_3:1_ for consistency). TE and ICC were calculated based on three trials conducted in the baseline condition. TE was determined by dividing the standard deviation (SD) of the differences from the three pre-condition trials by the square root of 2, using a 95% confidence interval (CI) (Hopkins, 2000), expressed as a coefficient of variation (CV). The interpretation of intraclass correlation coefficient (ICC) values was classified according to Koo and Li (2016) as follows: >0.9 (excellent), 0.75–0.9 (good), 0.5–0.75 (moderate), and <0.5 (poor). Data normality was assessed using the Shapiro-Wilk test, while the Levene’s test was employed to check for equality of variances. The Kruskal-Wallis non-parametric test was applied if the Levene’s test’s results indicated unequal variances (*p* < 0.05). Given that BLD did not follow a normal distribution (*p* < 0.05), analyses were conducted using the Friedman test with Conover’s post hoc test to compare CMJ metrics across the four successive bouts. An alpha level of 0.05 was set for all statistical analyses. For non-parametric evaluations, effect size (ES) was determined using Kendall's W (W), with classifications of agreement as follows: “no agreement” (W < 0.10), “weak agreement” (0.10 ≥ W < 0.30), “moderate agreement” (0.30 ≥ W < 0.60), “strong agreement” (0.60 ≥ W < 1), and “perfect agreement”. All statistical analyses were performed using JASP software (version 0.11.1, JASP Team, University of Amsterdam, Netherlands).

## Results

Table 1 displays the reliability values (ICC and TE with 95% confidence intervals) for the CMJ in all metrics, focusing on bilateral metrics, the sum of non-dominant and dominant legs, and BLD during the baseline condition. All variables showed excellent relative reliability (ICC > 0.75) for both limbs. In terms of absolute reliability (TE), most variables exhibited slightly higher values in the non-dominant limb compared to the dominant limb.

**Table 1:** Descriptive (Mean ± SD) and reliability values (ICC and TE) of CMJ metrics considering bilateral, sum of dominant and non-dominant legs and BLD in the baseline situation.

CMJ metrics	Mean ± SD	ICC_3,1_	TE
**Jump Height**			
Bilateral (cm)	40.5 ± 6.7	0.87 (0.67–0.95)	3.24 (2.44–4.90)
Sum of Legs (cm)	57.0 ± 10.3	0.95 (0.87–0.98)	2.69 (2.03–4.08)
BLD (%)	−27.7 ± 12.4	0.78 (0.51–0.92)	7.5 (5.67–11.3)
**Mean Power**			
Bilateral (W)	1939.5 ± 401.5	0.80 (0.54–0.93)	210.75 (159.22–319.36)
Sum of Legs (W)	2478.1 ± 418.2	0.97 (0.92–0.99)	82.97 (62.68–125.73)
BLD (%)	−20.5 ± 16.2	0.87 (0.67–0.95)	7.40 (5.59–11.21)
**Peak Power**			
Bilateral (W)	3726.9 ± 788.0	0.80 (0.54–0.93)	410.79 (310.33–622.47)
Sum of Legs (W)	4722.8 ± 807.3	0.97 (0.91–0.99)	172.41 (130.25–261.25)
BLD (%)	−19.8 ± 16.7	0.86 (0.66–0.95)	7.77 (5.87–11.78)
**Peak Force**			
Bilateral (N)	1757.6 ± 274.5	0.86 (0.65–0.95)	124.15 (93.79–188.13)
Sum of Legs (N)	3023.0 ± 480.6	0.98 (0.95–0.99)	71.51 (54.02–108.36)
BLD (%)	−41.5 ± 6.9	0.79 (0.51–0.92)	3.72 (2.81–5.64)
**Peak Velocity**			
Bilateral (m·s^−1^)	2.6 ± 0.3	0.76 (0.47–0.92)	0.18 (0.14–0.28)
Sum of Legs (m·s^−1^)	3.8 ± 0.5	0.98 (0.96–0.99)	0.07 (0.05–0.11)
BLD (%)	−31.8 ± 9.7	0.85 (0.64–0.95)	4.87 (3.68–7.38)
**Impulse**			
Bilateral (N·s)	276.7 ± 53.6	0.75 (0.44–0.91)	29.84 (22.54–45.21)
Sum of Legs (N·s)	381.0 ± 68.1	0.88 (0.70–0.96)	24.02 (18.15–36.40)
BLD (%)	−26.0 ± 15.0	0.94 (0.83–0.98)	4.52 (3.41–6.84)

Table 2 presents the absolute values of CMJ bilateral metrics at baseline (post-match 1, 2, 3 and 4). A substantial effect of successive bouts was found only in the impulse (*X*^2^_(4,48)_ = 23.20, *p* < 0.001, *W* = 0.44 [strong agreement]), with lower values at post-match 3 compared to post-match 1 (*p* = 0.004), and post-match 4 compared to post-match 2 (*p* < 0.011). No significant differences were observed across the simulated judo bouts for jump height (*X*^2^_(4,48)_ = 0.12, *p* = 0.99, *W* = 0.13; weak agreement), mean power (*X*^2^_(4,48)_ = 1.04, *p* = 0.90, *W* = 0.07; no agreement), peak power (*X*^2^_(4,48)_ = 2.27, *p* = 0.68, *W* = 0.04 [no agreement]), peak force (*X*^2^_(4,48)_ = 9.10, *p* = 0.058, *W* = 0.16 [weak agreement]), and peak velocity (*X*^2^_(4,48)_ = 5.23, *p* = 0.26, *W* = 0.10 [weak agreement]).

**Table 2:** Mean ± SD of the absolute values of CMJ bilateral metrics throughout four successive judo bouts.

CMJ metrics	Baseline	Post-match 1	Post-match 2	Post-match 3	Post-match 4
Jump Height (cm)	43.2 ± 6.3	43.1 ± 6.2	43.3 ± 7.0	43.2 ± 6.6	43.5 ± 6.5
Mean Power (W)	2101.4 ± 297.6	2123.7 ± 309.5	2086.8 ± 308.5	2115.1 ± 299.1	2102.3 ± 296.5
Peak Power (W)	4038.4 ± 577.7	4093.5 ± 622.4	4008.6 ± 594.9	4067.5 ± 578.1	4038.4 ± 577.7
Peak Force (N)	1883.1 ± 261.1	1922.5 ± 245.0	1899.9 ± 270.9	1869.0 ± 259.7	1880.5 ± 266.3
Peak Velocity (m·s^−1^)	2.69 ± 0.2	2.71 ± 0.1	2.68 ± 0.3	2.69 ± 0.2	2.69 ± 0.3
Vertical Impulse (N·s)	287.3 ± 37.6	304.2 ± 41.9	301.1 ± 47.8	270.5 ± 37.8*#	290.2 ± 46.5


* Different from baseline; # Different from post-match 2

Table 3 presents BLD indices for all CMJ metrics at baseline, post-match 1, post-match 2, post-match 3, and post-match 4. A significant effect of judo bouts was found only in the impulse BDL (*X*^2^_(4,48)_ = 17.72, *p* = 0.001, η_p_^2^ = 0.23; large), with higher values at post-match 3 compared to post-match 2 (*p* = 0.001) and post-match 4 (*p* < 0.001). No significant differences were found over the simulated judo bouts for BDL of jump height ( *X*^2^_(4,48)_ = 6.40, *p* = 0.17, η_p_^2^ = 0.13; small), mean power (*X* ^2^_(4,48)_ = 5.35, *p* = 0.25, η_p_^2^ = 0.07; small), peak power (*X*^2^_(4,48)_ = 7.63, *p* = 0.10, η_p_^2^ = 0.07; small), peak force (*X*^2^_(4,48)_ = 9.41, *p* = 0.052, η_p_^2^ = 0.16; medium), and peak velocity (*X*^2^_(4,48)_ = 3.44, *p* = 0.48, η_p_^2^ = 0.14; small).

**Table 3:** Mean ± SD of the BLD obtained in the CMJ metrics throughout four successive judo bouts.

BLD of CMJ metrics	Baseline	Post-match 1	Post-match 2	Post-match 3	Post-match 4
Jump Height (%)	−19.8 ± 4.3	−19.2 ± 4.6	−18.2 ± 7.3	−21.9 ± 5.9	−21.9 ± 4.7
Mean Power (%)	−13.2 ± 4.5	−12.3 ± 6.4	−12.3 ± 4.1	−13.1 ± 6.0	−14.3 ± 4.7
Peak Power (%)	−12.7 ± 5.0	−11.7 ± 6.7	−11.7 ± 4.8	−12.4 ± 6.5	−13.7 ± 5.0
Peak Force (%)	−37.0 ± 4.4	−35.5 ± 4.3	−36.2 ± 4.5	−37.5 ± 4.2	−37.1 ± 4.9
Peak Velocity (%)	−27.5 ± 2.8	−27.5 ± 2.8	−26.5 ± 3.4	−28.1 ± 3.6	−27.9 ± 2.4
Vertical Impulse (%)	−22.0 ± 6.0	−19.4 ± 10.0	−16.3 ± 8.8	−28.8 ± 9.8*$	−19.8 ± 11.8

* Different from post-match 2; $ Different from post-match 4

## Discussion

The current study aimed to investigate the effects of four successive judo bouts on BLD using the CMJ metrics. We supported our hypothesis, as BLD impulse increased from the third bout. Nevertheless, it is crucial to highlight that BLD of other jump variables did not change significantly over the bouts.

Our findings revealed that only the CMJ impulse decreased over the bouts (particularly after the third match). Similarly, only BLD of the impulse was affected after the third match, while no differences were found for the other metrics. BLD is a phenomenon where an athlete produces less combined force from both legs than the sum of the forces produced in each leg (Anders et al., 2021; Bracic et al., 2010; Železnik et al., 2022). This deficit is often associated with an inability to effectively coordinate or synchronize the force generated by each leg during movements like jumping (Bishop et al., 2021). The reduction in muscle activation linked to decreased force production has been identified as a likely contributor to the bilateral deficit (Howard and Enoka, 1991). In this sense, Vandervoort et al. (1984) suggest that bilateral deficit suppresses the recruitment of high-threshold fibers, which are essential for rapid force production. These fibers are known for their greater fatigue resistance (Schiaffino and Reggiani, 2011). Consequently, successive judo bouts may have influenced the fatigue state of these muscle fibers, potentially impacting BLD; however, this effect was only evident in the impulse metric.

Impulse in the vertical jump is associated with athletic performance in sports that demand explosive and powerful movements (Ruddock and Winter, 2015; Winter, 2005). In a fatigue situation, as observed in the CMJ impulse over bouts, muscle contractile capabilities are reduced, and force production is decreased (Gibson et al., 2001). As a result, the athlete may be unable to apply as much force to the ground quickly during the push-off phase in the CMJ, resulting in a decrease in impulse (I = F.t) (Winter, 2005). Considering the impulse-momentum theorem, the velocity attained at the center of mass at the end of the push-off, and thus, the resulting jump height, is directly influenced by the mechanical impulse generated in the direction of the movement (Kirby et al., 2011; McBride et al., 2010). Thus, it was expected that other jump variables correlated with impulse (e.g., peak velocity and jump height) would also demonstrate the same behavior; however, in our study, only impulse was sensitive enough to detect fatigue over bouts.

In a previous study, Bonitch-Domínguez et al. (2010) found no significant differences in peak power, force or velocity during a bilateral squat test across four successive judo bouts. Similarly, Kons et al. (2021) reported no difference in unilateral CMJ performance after four successive judo bouts, but did observe decreased handgrip strength performance and increased BLD in the handgrip test. This could suggest that the upper limbs were more affected by fatigue, consequently leading to a higher imbalance in the unilateral handgrip values. Additionally, neural mechanisms may play a role in maintaining force output during unilateral and bilateral CMJ testing while also protecting the limbs from potential injury caused by the imposed load (Gibson et al., 2001). In terms of judo-specific performance, these results suggest that higher BLD values in impulse may be linked to poorer performance in judo throwing techniques, particularly those requiring powerful movements and involving the stretch-shortening cycle (Kons et al., 2022), which are affected by acute fatigue (Detanico et al., 2015; Kons et al., 2021). This may also highlight the sensitivity of the measurements, with only impulse showing the ability to detect such changes. However, further research is needed to investigate this hypothesis and gain a more comprehensive understanding of BLD behavior under fatigued conditions.

Finally, the reliability of the CMJ metrics and BLD in the baseline condition was confirmed, suggesting that judo athletes were highly familiar with the CMJ test, as previously noted (Kons et al., 2021). Although BLD typically exhibits greater variability than other metrics (Atkinson and Nevill, 1998; Koo and Li, 2016), the values still demonstrated acceptable reliability in our study. Interestingly, the impulse BLD showed the highest reliability (ICC: 0.94), indicating that calculating BLD from the impulse metric may provide a reproducible alternative for CMJ assessment. This suggests that BLD derived from impulse data exhibits less signal variability on the force plate compared to other CMJ metrics, which may have facilitated the detection of differences in mechanisms related to BLD across bouts. The impulse is sensitive to detecting fatigue across successive bouts, possibly due to its ability to capture dynamic force production changes with minimal noise. Unlike other metrics, such as jump height or power output, impulse may be more closely linked to underlying neuromuscular fatigue mechanisms. The importance of conducting reliability analysis using ICC and TE lies in ensuring the consistency and accuracy of CMJ metrics (Bishop et al., 2023). Identifying the reliability of BLD is essential, as it informs about the accuracy of this index, particularly since it is derived from equations based on both bilateral and unilateral values. While metrics such as power output (mean and peak) and jump height are widely accepted as reliable in CMJ assessments (Koo and Li, 2016; Atkinson and Nevill, 1998), our findings indicate that BLD in the CMJ also serves as a reliable metric.

One potential limitation of our study is that we focused on simulated judo bouts, as evaluating athletes during actual competitions presents significant challenges. Nevertheless, we made efforts to closely mimic real match conditions by adhering to official match duration, rest intervals, and weight categories. For future research, we suggest employing more controlled tasks rather than simulated bouts to improve sensitivity in detecting changes in BLD. Furthermore, incorporating a complementary analysis of kinematic variables, such as knee and hip joint angles and squat depth, could provide deeper insights into motor control under fatigue conditions. Additionally, analyzing electromyography data could help identify key neuromuscular factors associated with BLD and the impact of fatigue.

## Conclusions

Our findings suggest that four consecutive judo bouts induced fatigue in the lower limbs, affecting BLD, with a significant impact observed primarily in the CMJ impulse metric. This indicates that the ability to generate force quickly in the lower limbs may be compromised, potentially leading to a decline in an athlete's overall performance during competition. However, the limited number of significant results and the lack of other BLD measures suggest that further research with a larger sample size and additional metrics is needed to strengthen these conclusions and provide a more comprehensive perspective considering the effects of successive bouts on neuromuscular aspects in judo athletes.
